# Isolation and characterization of H4N6 avian influenza viruses from mallard ducks in Beijing, China

**DOI:** 10.1371/journal.pone.0184437

**Published:** 2017-09-06

**Authors:** Junyi Hu, Xinyi Xu, Chenxi Wang, Guoxia Bing, Honglei Sun, Juan Pu, Jinhua Liu, Yipeng Sun

**Affiliations:** 1 Key Laboratory of Animal Epidemiology of the Ministry of Agriculture, College of Veterinary Medicine, China Agricultural University, Beijing, China; 2 China Animal Disease Control Center, Beijing, China; Sun Yat-Sen University, CHINA

## Abstract

The novel H7N9 influenza virus, which has caused severe disease in humans in China, is a reassortant with surface genes derived from influenza viruses in wild birds. This highlights the importance of monitoring influenza viruses in these hosts. However, surveillance of influenza virus in wild birds remains very limited in China. In this study, we isolated four H4N6 avian influenza viruses (AIVs) from mallard ducks in Beijing Wetland Park, which is located on the East Asia–Australasia migratory flyway. The gene segments of these Chinese H4N6 viruses were closest to AIVs in wild birds from Mongolia or the Republic of Georgia, indicating the interregional AIV gene flow among these countries. All of our isolates belonged to a novel genotype that was different from other H4N6 viruses isolated in China. We further evaluated the virulence and transmission of two representative H4N6 strains in mammalian models. We found that both of these H4N6 viruses replicated efficiently in mice without adaptation. Additionally, these two strains had a 100% transmission rate in guinea pigs via direct contact, but they had not acquired respiratory droplet transmissibility. These results reveal the potential threat to human health of H4N6 viruses in migratory birds and the need for enhanced surveillance of AIVs in wild birds.

## Introduction

Influenza viruses circulating in animals are novel to the human immune system; these viruses therefore pose a potential threat to public health once they are transmitted to humans. Moreover, animal influenza viruses can even cause pandemics in a human population once they acquire efficient transmissibility between people. Historically, influenza pandemics have been caused by animal influenza viruses (as in the 1918 and 2009 pandemics) or avian influenza viruses reassorting with human influenza viruses (pandemics in 1968 and 1957) [1–2]. Recently, a novel H7N9 influenza virus caused a total of 1364 laboratory-confirmed cases of influenza, with a nearly 33% case-fatality rate reported to the World Health Organization since its first detection in February 2013 in China [3]. Genetic analyses have found that this virus is a reassortant whose surface genes are directly derived from H7 and N9 subtypes of the wild bird viral gene pool, and with internal genes from H9N2 avian influenza viruses (AIVs) in chickens [4]. The novel H7N9 outbreak reminds us that other AIV subtypes in wild birds also pose a threat to humans, either alone by reassortment with other influenza viruses. Therefore, it is important to rigorously monitor the prevalence and biological properties of AIVs in wild birds.

The H4N6 AIV subtype circulates among waterfowl worldwide, including in Asian, European, African, and North American countries [5–6]. It has been noted that H4N6 viruses have been repeatedly found in mammalian species, such as humans and pigs [7–11]. H4N6 is frequently isolated from domestic ducks in China [12–14]. Genetic analysis indicates that these H4N6 viruses are undergoing complex and frequent reassortment events. In particular, they can efficiently replicate in the respiratory tissues of infected mice and spread among guinea pigs via direct transmission. Moreover, some strains have acquired respiratory droplet transmissibility in a guinea pig model [12]. Although the characteristics of H4N6 in domestic ducks in China have been well studied, the genetic and biological properties of H4N6 in wild birds of this region have not been reported.

In April 2016, we isolated four H4N6 AIVs from mallard ducks in Beijing Wild Duck Lake Wetland Nature Reserve, which is located on the East Asia–Australasia migratory flyway. The genomes of these viruses were sequenced and analyzed against all H4N6 viruses in China, available from the NCBI Influenza Virus Sequence Database. To evaluate their potential threat to human health, we further determined the replication and transmission of two H4N6 strains in mouse and guinea pig models.

## Materials and methods

### Sampling methods and ethical compliance

We collected the fresh fecal samples from the ground after the wild birds flew away without direct contact with any birds. Feces were collected from the mallard ducks in Beijing Wild Duck Lake Wetland Nature Reserve (40°22′04"~40°30′31"N, 115°46′16"~115°59′48"E). The study and sampling procedures were permitted by Management Office of Yanqing Country Wild Duck Lake Wetland Nature Reserve.

All animal work was approved by the Beijing Association for Science and Technology (approval SYXK [Beijing] 2007–0023) and conducted in accordance with Beijing Laboratory Animal Welfare and Ethics guidelines, as issued by the Beijing Administration Committee of Laboratory Animals, and in accordance with China Agricultural University (CAU) Institutional Animal Care and Use Committee guidelines (SKLAB-B-2010-003). Mice and guinea pigs were humanely euthanized using anesthesia followed by cervical dislocation. All animal researches in this study adhere to the ARRIVE Guidelines. A completed ARRIVE guidelines checklist is included in S1 Checklist.

### Virus isolation

We collected feces from mallard ducks in Beijing Wetland Park Randomly. Viruses were isolated by inoculation in 10-day old specific-pathogen-free (SPF) chicken eggs, as previously described [15]. After incubation at 37°C for 48 h, the allantoic fluid was harvested and identified serologically with reference antisera to each known influenza virus [16].The viruses were purified by plaque assay in MDCK cells and no other subtype influenza viruses were isolated in the samples.

### Sequence analyses

RNA extraction was performed using an RNeasy Mini Kit (QIAGEN, Valencia, CA, USA), according to the manufacturer’s instructions. Reverse transcription was performed using Uni12 primer to generate cDNA [17] and amplified by PCR with specific primers for each viral gene segment. PCR products of all eight segments of these viruses were gel-purified using the QIAquick PCR purification kit (QIAGEN) and then sequenced by TSINGKE Biological Technology Co., China.

We used a sequence analysis software package (DNASTAR, Inc., Madison, WI, USA), and multiple sequence alignment was carried out using ClustalW [18]. Phylogenetic and molecular evolutionary analyses were conducted using the MEGA6.0 software package, by the neighbor-joining method. Tree topology was evaluated using neighbor-joining methodwith 1000 bootstrap replicates [19], with a distinct phylogenetic lineage and bootstrap support values ≥80% indicating a common origin.

### Definition of genotypes

The genotype classification was defined based on the topologies of the phylogenetic trees as described previously [20–23]. In detail, the genotypes of the viruses were determined by the combination of lineage assignments of each of the eight segments based on the gene phylogeny analysis. A genotype was defined when the phylogenetic lineages of the eight genes resulted in a unique gene constellation.

### Studies in mice

Groups of nine 6-week-old female BALB/c mice (Vital River Laboratories, Beijing, China) were lightly anesthetized with Zoletil 50 (Zoletil; Virbac SA, Carros, France), and then inoculated intranasally (i.n.) with 50 μl of allantoic fluid containing the isolated H4 influenza viruses at 10^6^ EID_50_. At 3 and 5 days post-inoculation (dpi), three of the nine inoculated mice in each group were euthanized and their organs, including heart, liver, spleen, lung, kidneys, and brain were collected under sterile conditions for virus titration. Embryonated chicken eggs were used to determine the EID_50_s of the supernatants, using the method of Reed and Muench [24]. The other three mice from each group were monitored daily for 14 days for weight loss [25].

### Studies in guinea pigs

Female Hartley strain guinea pigs weighing between 300 and 350 g(Vital River Laboratories, Beijing, China), serologically negative for influenza viruses, were used. The guinea pig studies were performed as described in detail previously [12] with minor revision. Briefly, they were anesthetized with Zoletil 50 by intramuscular injection. For the direct contact transmission experiment, three guinea pigs per group were housed in a cage inside an isolator and inoculated i.n. with 10^6^ EID_50_ of each virus. 24 hours later, the inoculated guinea pigs were co-housed with three naive ones. Nasal washes were collected at 2-day intervals beginning at 2 dpi (1 day after contact) and were titrated in eggs.

For the respiratory droplet transmission experiment, groups of three guinea pigs were inoculated i.n. with 10^6^ EID_50_ of each virus. The next day, each guinea pig was transferred to a specially designed cage, and was paired with a naive animal that was housed in an adjacent cage (5 cm away), separated by a double-layered net divider that allowed horizontal airflow from the inoculated to the naive animals. Nasal washes were collected and titrated as described in the direct contact transmission experiment. Sera were collected from all animals at 21 dpi for HI antibody detection.

### Nucleotide sequence accession numbers

The nucleotide sequences of the four H4N6 viruses isolated in this study have been deposited in GenBank under accession numbers from MF144938 to MF144969.

## Results

We isolated four H4N6 influenza viruses from the feces of mallard ducks in a wetland reserve in Beijing, China, in April 2016. The positive rate was 13.3%. BLAST searches in GenBank (http://blast.ncbi.nlm.nih.gov/Blast.cgi) for sequences most similar to those of the isolates showed that the PB2 gene had a close relationship to A/duck/Mongolia/572/2015 (H3N8), with nucleotide identity of 99%. PB1, PA, HA, NA, and M were closest to A/duck/Mongolia/543/15 (H3N8), with identities of 99%. NP was closest to A/mallard/Republic of Georgia/13/2011 (H6N2), with 98% identity, and NS was closest to A/common shelduck/Mongolia/2076/2011 (H3N8), with identity of 99%. The genomes of these H4N6 isolates were also analyzed against all 38 Chinese H4N6 viruses with eight gene sequences in the NCBI viral sequence database.

### Phylogenetic analysis of H4N6 genes

**Fig 1:**
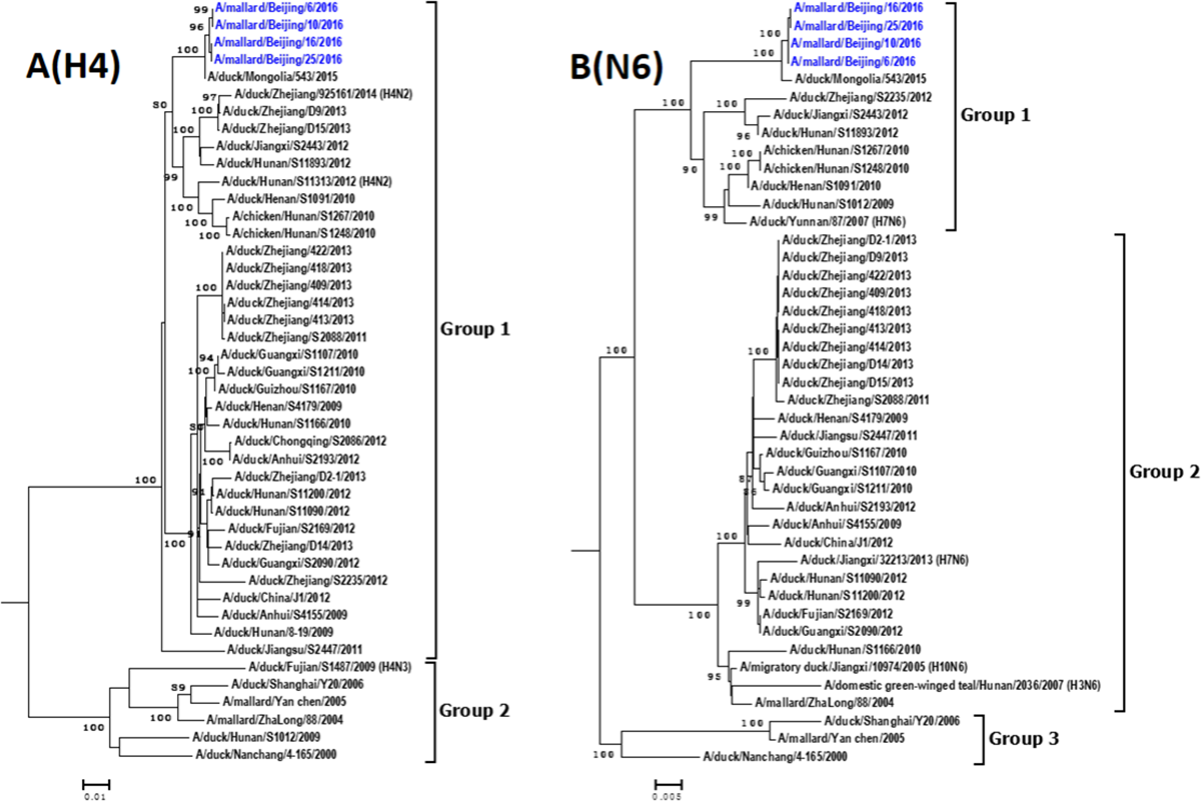
**Phylogenetic trees of H4 (A) and N6 (B) genes of H4N6 AIVs in China.** Phylogenetic trees were generated by the neighbor-joining method and bootstrapped with 1000 replicates using MEGA6. Analysis was based on nucleotides 1–1693 of the HA gene and 1–1413 of the NA gene. Virus subtypes that are not H4N6 are indicated in brackets. Virus strains isolated in this study are highlighted in red.

Phylogenetic analysis of the HA gene of H4N6 influenza viruses isolated in China during 2000–2016 indicate that all HA genes of the H4N6 viruses in China belong to the Eurasian lineage and can be divided into two groups (Fig 1A). The different clades are related to the year of isolation. H4N6 strains isolated after 2009 belong to group 1 whereas five isolates from 2000 to 2009 fall into group 2. The homology with other H4N6 AIVs of the HA genes of the four H4N6 viruses isolated in this study was below 99%; they were most closely related to an H4N6 virus in Mongolia (A/duck/Mongolia/543/2015). The intragroup homology of HA gene was over 92.9%. The phylogenetic tree of the NA gene of H4N6 AIVs isolated in China includes three groups (Fig 1B). Except for three early H4N6 isolates belonging to group 3, all other Chinese H4N6 viruses fall into group 1 or group 2. Our isolates were in group 1. The NA genes shared nucleotide sequence identities of over 90.9%.

**Fig 2:**
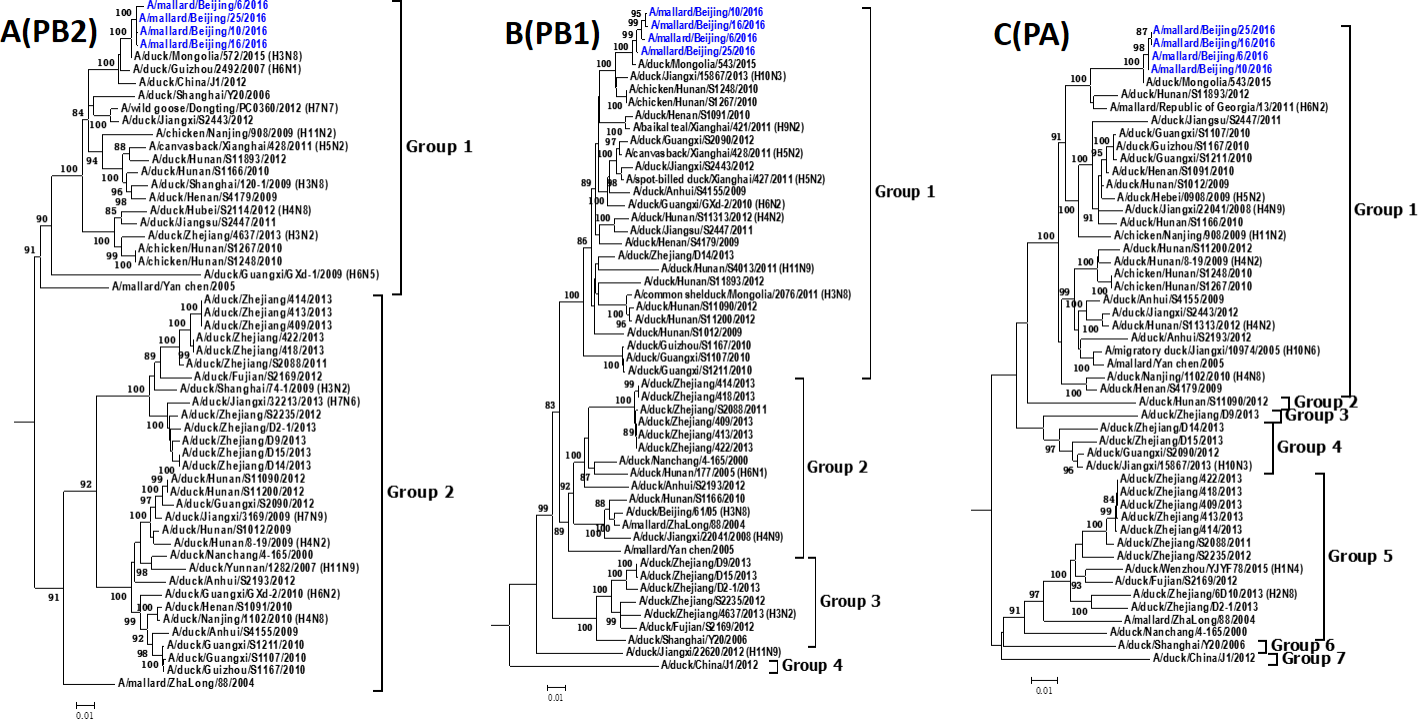
**Phylogenetic trees of PB2 (A), PB1 (B) and PA (C) genes of H4N6 AIVs in China.** Phylogenetic trees were generated by the neighbor-joining method and bootstrapped with 1000 replicates using MEGA6. Analysis was based on nucleotides 1–2277 of the PB2 gene, 1–2274 of the PB1 gene, and 1–2151 of the PA gene. Virus subtypes that are not H4N6 are indicated in brackets. Virus strains isolated in this study are highlighted in red.

**Fig 3:**
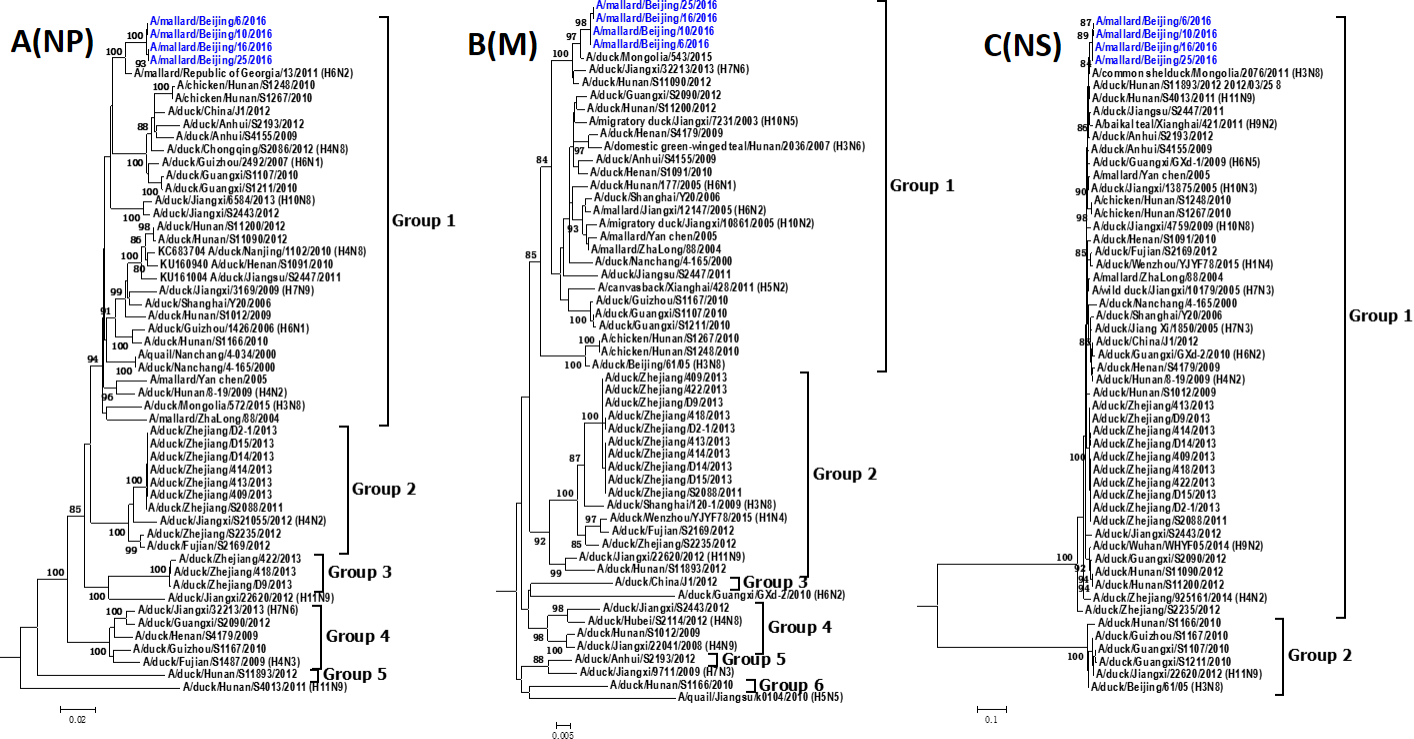
**Phylogenetic trees of NP (A), M (B), and NS (C) genes of H4N6 AIVs in China.** Phylogenetic trees were generated by the neighbor-joining method and bootstrapped with 1000 replicates using MEGA6. Analysis was based on nucleotides 1–1497 of the NP gene, 1–982 of the M gene, and 1–838 of the NS gene. Virus subtypes that are not H4N6 are indicated in brackets. Virus strains isolated in this study are highlighted in red.

Phylogenetic analysis indicated that H4N6 AIVs that belong to the same group of surface genes usually fall into different groups of internal genes (Figs 2 and 3), indicating that reassortment events among these viruses are frequent. The phylogenic trees of the PA gene segment of 38 AIVs in China is mostly multiple which includes seven groups. NS genes of H4N6 AIVs are divided into group 1 and group 2, which belong to allele A and allele B, respectively [26]. Most H4N6 AIVs (92.1%), including our H4N6 strains, belong to group 1. The intragroup homology of the PB2, PB1, PA, NP, M, NS was over 89.3%, 93.9%, 93.3%, 93.0%, 96.1% and 95.6%, respectively.

### Genotyping

**Fig 4:**
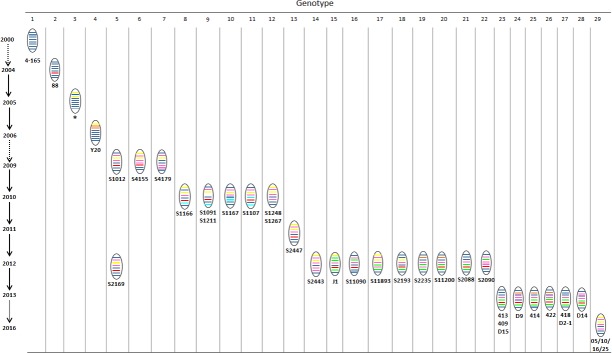
The genotypic evolution of H4N6 AIVs in China from 2000 to 2016. The eight gene segments listed are (top to bottom): PB2, PB1, PA, HA, NP, NA, M, and NS genes. The colors of the eight gene segments of the first isolate (A/duck/Nanchang/4-165/2000) were defined to be the same, and a new color represent a different lineage from this strain. The abbreviation of virus which possessed the corresponding genotype are listed below the genotype. The gene constellation for different genotypes, the viruses that possessed these, and the abbreviation of virus name are given in S1 Table.

Based on the phylogenetic diversity, we divided the 38 Chinese H4N6 AIVs into 29 genotypes. The genotypic evolution of H4N6 AIVs isolated in China from 2000 to 2016 is described in Fig 4 and summarized in S1 Table. The genotypes of these H4N6 viruses were very multiple, and only H4N6 AIVs isolated in similar regions and at approximately the same time belonged to the same or similar genotype(s). However, since available H4N6 isolates are very few at present, whether this is a characteristic of H4N6 AIVs need to be evaluated by further epidemic surveillance. Additionally, novel genotypes were identified every year, implying that reassortment occurred frequently.

**Fig 5:**
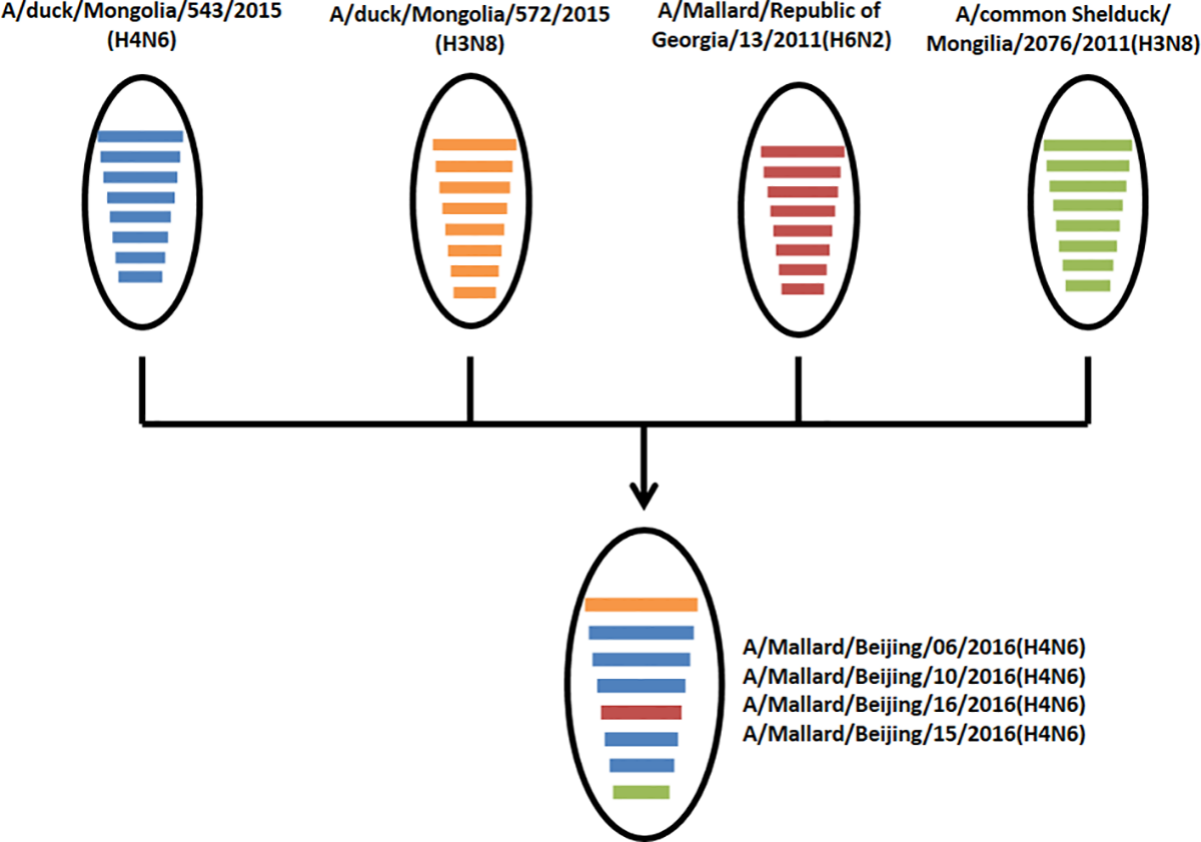
Schematic representation of putative genomic compositions of the four H4N6 AIVs isolated in this study and their possible parent viruses. The eight gene segments (from top to bottom) in each virus are PB2, PB1, PA, HA, NP, M, and NS. Each color represents a separate virus background. The simplified schematic illustration is based on nucleotide-distance comparison and phylogenetic analysis.

The four H4N6 viruses isolated in this study shared a genotype that was different from all the other Chinese H4N6 AIVs. This genotype is similar to those of A/duck/Mongolia/543/15 (H4N6), but reassorted with A/duck/Mongolia/572/15 (H3N8), A/mallard/Republic of Georgia/12/11 (H6N2), and A/common shelduck/Mongolia/2076/11 (H3N8) for the PB2, NP, and NS genes, respectively (Fig 5). This indicates that this reassortment occurred during the migration of wild birds, but not in China.

### Molecular characterization

All of our H4N6 strains possessed a single basic amino acid (arginine) in the HA cleavage site, the signature of low-pathogenicity AIVs. The receptor binding site of influenza virus HA is formed by the 190-helix, 130-loop, and 220-loop at its globular head. The HA receptor binding site of all four viruses in this study was highly conserved, containing 98Y, 134G, 135K, 136S, 137G, 138A, 153W, 155V, 183H, 190E, 194L, 195Y, 224R, 225G, 226Q, 227S, 228G, and 229R (H3 numbering, which is used throughout this work). None of these residues have been reported to be involved in the recognition of human-type receptors. All of these H4N6 AIVs had five conserved potential glycosylation sites on HA at positions 6 to 8, 22 to 24, 165 to 167, 296 to 298, and 483 to 485. There were no deletions in the NA genes of any of these viruses. The His274Tyr mutation associated with oseltamivir and zanamivir was not observed in NA.

PB2-627K and -701N are known to play an important role in the virulence and transmission of influenza viruses in mammals. None of these known mutations were observed in the H4N6 AIVs isolated in this study. F26, A/I27, T/V30, N31, E34, and F38 in the M2 protein are amantadine resistance markers [27]. No such substitutions were found in our H4N6 AIVs.

### Pathogenicity and replication in mice

**Fig 6:**
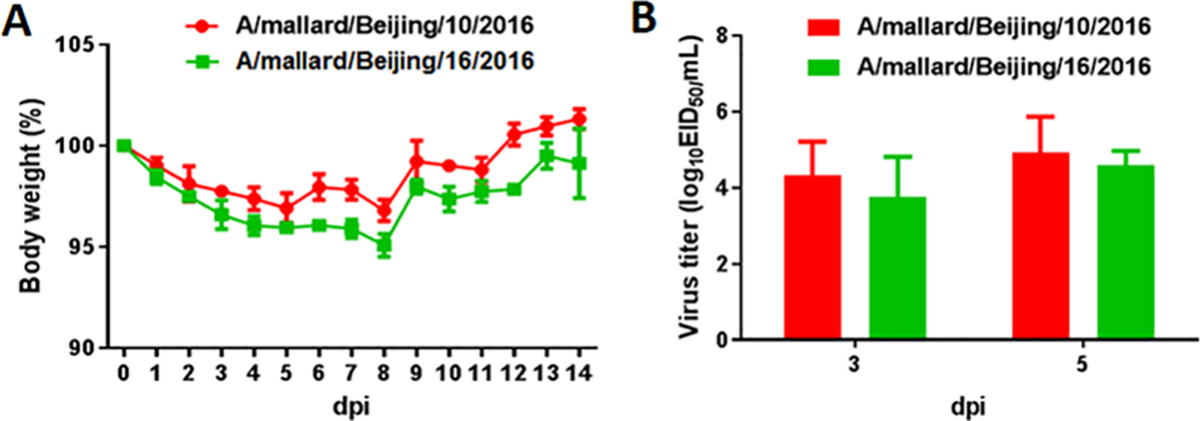
Weight loss and lung virus titration in mice inoculated with H4N6 AIVs. Six-week-old female BALB/c mice (n = 3 mice/group) were inoculated i.n. with 10^6^ EID_50_ of virus. The body weights of inoculated mice were measured daily and are represented as percentages of weight on the day of inoculation (day 0). The averages for each group are shown (A).The lungs of each mouse in each group were collected at 3 and 5 dpi, respectively, for virus titration (B).

According to the genetic relationship, we selected two representative H4N6 influenza viruses that we isolated in Beijing and evaluated their pathogenicity and replication in BALB/c mice. Most weight loss occurred in mice infected with A/mallard/Beijing/10/2016 and A/mallard/Beijing/16/2016, with 2.8% ± 1.2% and 4.2% ± 0.9%, respectively, which occurred during 8–9 dpi (Fig 6A). These viruses replicated efficiently in the lungs of infected mice, with virus titers of 4.4 ± 0.9 and 3.8 ± 0.8 log_10_EID_50_/mL at 3 dpi, and 5.3 ± 0.8 and 4.6 ± 0.4 log_10_EID_50_/mL at 5 dpi (Fig 6B). No virus was detected in the heart, liver, spleen, kidneys, or brain of any mice.

### Transmission in guinea pigs

**Fig 7:**
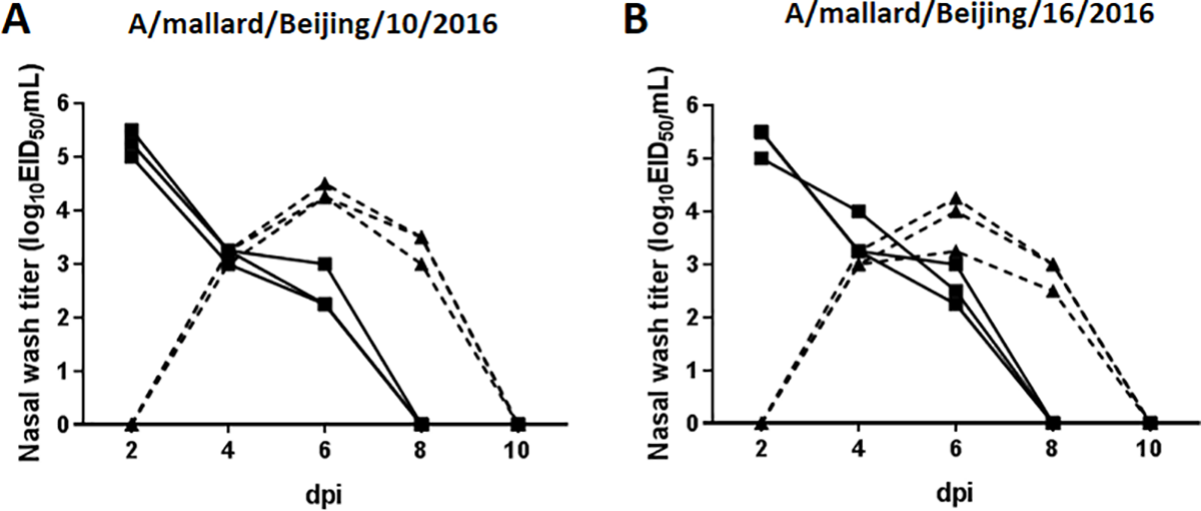
Contact transmission of H4N6 AIVs in guinea pigs. Three guinea pigs were intranasally inoculated with 10^6^ EID_50_ of A/mallard/Beijing/10/2016 (A) or A/mallard/Beijing/16/2016 (B) viruses. At 24 hpi, the inoculated guinea pigs were placed in a cage with three naive guinea pigs. Nasal wash titers are plotted as a function of time post-inoculation. Titers of intranasally inoculated animals are represented by solid lines and filled squares; titers of exposed guinea pigs are shown with dashed lines and filled triangles.

To evaluate the transmissibility of these viruses among mammals, we intranasally inoculated three guinea pigs with 10^6^ EID_50_ of A/mallard/Beijing/10/2016 or A/mallard/Beijing/16/2016. At 24 hours post-inoculation (hpi), the inoculated guinea pigs were placed in a cage with three naive guinea pigs. Nasal washes were collected from all animals and viruses were titrated by EID_50_ infectivity assay. As shown in Fig 7, both A/mallard/Beijing/10/2016 and A/mallard/Beijing/16/2016 were detected at 2, 4, and 6 dpi, with peak titers of 5.1 ± 0.1 and 5.3 ± 0.3 log_10_EID_50_/mL, respectively. These viruses were able to transmit to all of the contact animals, which themselves shed viruses from 4 to 8 dpi. Peak titers occurred in contact guinea pigs at 6 dpi, with 4.4 ± 0.1 and 3.4 ± 0.1 log_10_EID_50_/mL for A/mallard/Beijing/10/2016 and A/mallard/Beijing/16/2016, respectively.

We further evaluated respiratory droplet transmission among guinea pigs. Three animals were inoculated intranasally with 10^6^ EID_50_ of each virus and then housed individually in solid stainless steel cages in an isolator. At 24 hpi, three naive guinea pigs were each placed in a cage adjacent to that of an inoculated animal. Each pair of animals (one inoculated and one exposed) was placed 5 cm apart and separated by a double-layered net divider. No virus shedding or seroconversion was detected in any of the exposed guinea pigs (data not shown). These results indicate that A/mallard/Beijing/10/2016 and A/mallard/Beijing/16/2016 viruses can transmit efficiently by direct contact among guinea pigs but have not acquired respiratory droplet transmissibility, in this animal model.

## Discussion

In the present study, we isolated four H4N6 AIVs from wild mallard ducks in Beijing. Phylogenetic analyses demonstrated that all these isolates were of the Eurasian lineage. The genotype of these AIVs was different from that of other H4N6 viruses in China or in other countries, and it has not been identified previously. The isolated viruses replicated efficiently in the respiratory system of mice and guinea pigs without adaptation. Our isolated H4N6 viruses were able to transmit to all naive guinea pigs via direct contact but had not acquired respiratory droplet transmissibility between guinea pigs in our study. This study stresses the need for continued monitoring of AIVs in wild birds, to gain a better understanding of the emergence of strains with the potential to infect humans.

Phylogenetic analyses of HA genes indicated that, like all of the H4N6 AIVs previously isolated in China, the four H4N6 AIVs isolated in this study belonged to the Eurasian lineage. However, these viruses fell into groups different from the other Chinese H4N6 AIVs, which were genetically closest to an H4N6 AIV isolated in Mongolia (A/duck/Mongolia/543/2015). The other seven gene segments of the four H4N6 AIVs were also closest to those in other countries, including H3N8 virus A/duck/Mongolia/543/2015 (PB1, PA, NA, and M), H3N8 virus A/duck/Mongolia/572/2015 (PB2), H6N2 virus A/mallard/Republic of Georgia/13/2011 (NP), and H3N8 virus A/common shelduck/Mongolia/2076/2011 (NS). These results suggest that our H4N6 strains were introduced from other countries. Additionally, the genotype of these viruses belonged to a novel genotype, which might be the result of reassortment events occurring during the migration of wild birds.

The HA receptor binding sites of the four H4N6 AIVs isolated in this study have not been reported to be involved in the recognition of human-type receptors. Additionally, mutations related to the enhanced virulence and transmission of influenza viruses in mammals, including PB2-627K and -701N, were also not observed in any of the four isolated viruses. H4N6 strains previously isolated from domestic ducks in China also did not possess these mutations related to mammalian adaptation [13,14,28]. Hossain et al. demonstrated that duck influenza virus could acquire mammalian-associated genetic changes after adaptation in land-based birds, including chickens and quail [29]. H4N6 AIVs have not become mammalian-associated because they have been circulating in waterfowl. However, once they are transmitted to land-based birds, they might be more easily adapted to mammals. It has been noted that some H4N6 AIVs have been isolated in chickens, according to sequences in the NCBI Influenza Virus Sequence Database. Therefore, it is necessary to monitor H4N6 AIVs in land-based birds. Because our four H4N6 viruses have been circulating in wild birds, no mutations associated with drug-resistance were found in these strains. By contrast, V27I, which is associated with amantadine resistance, has been frequently identified in H4N6 AIVs isolated from domestic ducks, which might induced by drug use [14,28].

The maximum body weight loss in mice infected with A/mallard/Beijing/10/2016 and A/Mallard/Bejing/16/2016 was 2.8% and 4.2%, respectively. Weight loss in ducks infected with H4N6 ranges from 0% to 13%, as reported previously [28]. A/mallard/Beijing/10/2016 and A/mallard/Beijing/16/2016 replicate efficiently in the lungs of mice and the virus titers are similar to those of H4N6 AIVs isolated from domestic ducks [14,28]. Guinea pig models are useful for evaluating the potential threat of influenza virus to human health [29]. The nasal tract of guinea pigs contains both SAα2,6-Gal and SAα2,3-Gal receptors; this is more similar to the human respiratory system as compared with mice, whose upper respiratory system mainly contains SAα2,3-Gal receptors [29,30]. In the present study, the H4N6 viruses isolated from wild birds were shed at high titers from the nasal tracts of guinea pigs and had 100% transmissibility among guinea pigs. However, our findings showed that these H4N6 AIVs had not acquired respiratory droplet transmissibility between guinea pigs. Liang et al. demonstrated that some H4N6 AIVs isolated in domestic ducks could be transmitted to one or two of five exposed guinea pigs via respiratory droplets [28]. The increased respiratory droplet transmissibility of H4N6 AIVs in domestic ducks compared with those in mallard ducks might be the result of adaptation of the viruses in domestic ducks, or this might be owing to these AIVs evolving in domestic ducks from another H4N6 lineage or genotype. Nevertheless, the results of animal studies indicate that H4N6 AIVs in wild birds readily infect mammals without adaptation and could efficiently spread among guinea pigs by direct contact. Therefore, the potential threat to human health warrants continued monitoring of these viruses.

China is regarded as an epicenter of pandemic influenza viruses, as a region where different AIVs co-circulate. AIVs in wild birds frequently reassort with those in domestic birds, resulting in novel reassortant influenza viruses. If the HA gene originates from AIVs in wild birds, the avian influenza vaccines used in commercial poultry might be unable to protect them and these AIVs can spread rapidly and even be transmitted to humans. In recent years, novel H7N9 and H10N8 influenza outbreaks in humans in China were all owing to reassortant viruses derived from AIVs in wild and domestic birds.

Here, we found H4N6 AIVs with a novel genotype among wild birds in China. These viruses were found to readily replicate and transmit among mammals. Therefore, it is important to maintain continued monitoring of AIVs in wild birds in China, to aid in the prediction and prevention of human influenza pandemics.

## Supporting information

S1 Checklist Completed ‘‘The ARRIVE guidelines checklist” for reporting animal data in this study. (DOCX)

S1 Table H4N6 AIVs with different genotypes in China from 2000 to 2016. (DOCX)

## References

[1] Richard M, de Graaf M, Herfst S. Avian influenza A viruses: from zoonosis to pandemic. Future Virol. 2014;9(5):513–24. doi: 10.2217/fvl.14.30 25214882 10.2217/fvl.14.30 PMC4157675

[2] Gao R, Cao B, Hu Y, Feng Z, Wang D, Hu W, et al Human infection with a novel avian-origin influenza A (H7N9) virus. NEJM. 2013;368(20):1888–97. doi: 10.1056/NEJMoa1304459 23577628 10.1056/NEJMoa1304459

[3] Human infection with avian influenza A (H7N9) virus-China. World Health Organization. 2017;4: 5 Available from: Http://www.who.int.

[4] Lam TT, Wang J, Shen Y, Zhou B, Duan L, Cheung CL, et al The genesis and source of the H7N9 influenza viruses causing human infections in China. Nature. 2013;502(7470):241–4. doi: 10.1038/nature12515 23965623 10.1038/nature12515 PMC3801098

[5] Simulundu E, Ishii A, Igarashi M, Mweene AS, Suzuki Y, Hang'ombe BM, et al Characterization of influenza A viruses isolated from wild waterfowl in Zambia. J Gen Virol. 2011;92(Pt 6):1416–27. doi: 10.1099/vir.0.030403-0 21367986 10.1099/vir.0.030403-0

[6] Nguyen DC, Uyeki TM, Jadhao S, Maines T, Shaw M, Matsuoka Y, et al Isolation and characterization of avian influenza viruses, including highly pathogenic H5N1, from poultry in live bird markets in Hanoi, Vietnam, in 2001. J Virol. 2005;79(7):4201–12. doi: 10.1128/JVI.79.7.4201-4212.2005 15767421 10.1128/JVI.79.7.4201-4212.2005 PMC1061558

[7] Karasin AI, Brown IH, Carman S, Olsen CW. Isolation and characterization of H4N6 avian influenza viruses from pigs with pneumonia in Canada. J Virol. 2000;74(19):9322–7. 10982381 10.1128/jvi.74.19.9322-9327.2000 PMC102133

[8] Ninomiya A, Takada A, Okazaki K, Shortridge KF, Kida H. Seroepidemiological evidence of avian H4, H5, and H9 influenza A virus transmission to pigs in southeastern China. Vet Microbiol. 2002;88(2):107–14. 12135631 10.1016/s0378-1135(02)00105-0

[9] Kayali G, Ortiz EJ, Chorazy ML, Gray GC. Evidence of previous avian influenza infection among US turkey workers. Zoonoses Public Hlth. 2010;57(4):265–72. 10.1111/j.1863-2378.2009.01231.x 19486492

[10] Kayali G, Barbour E, Dbaibo G, Tabet C, Saade M, Shaib HA, et al Evidence of infection with H4 and H11 avian influenza viruses among Lebanese chicken growers. PloS One. 2011;6(10):e26818 doi: 10.1371/journal.pone.0026818 22046370 10.1371/journal.pone.0026818 PMC3203926

[11] Su S, Qi W, Chen J, Zhu W, Huang Z, Xie J, et al Seroepidemiological evidence of avian influenza A virus transmission to pigs in southern China. J Clin Microbiol. 2013;51(2):601–2. doi: 10.1128/JCM.02625-12 23175250 10.1128/JCM.02625-12 PMC3553921

[12] Liang L, Deng G, Shi J, Wang S, Zhang Q, Kong H, et al Genetics, Receptor Binding, Replication, and Mammalian Transmission of H4 Avian Influenza Viruses Isolated from Live Poultry Markets in China. J Virol. 2015;90(3):1455–69. doi: 10.1128/JVI.02692-15 26581996 10.1128/JVI.02692-15 PMC4719592

[13] Wu H, Peng X, Peng X, Cheng L, Lu X, Jin C, et al Genetic characterization of natural reassortant H4 subtype avian influenza viruses isolated from domestic ducks in Zhejiang province in China from 2013 to 2014. Virus Genes. 2015;51(3):347–55. doi: 10.1007/s11262-015-1245-2 26350888 10.1007/s11262-015-1245-2

[14] Shi Y, Cui H, Wang J, Chi Q, Li X, Teng Q, et al Characterizations of H4 avian influenza viruses isolated from ducks in live poultry markets and farm in Shanghai. Sci Rep. 2016;6:37843 doi: 10.1038/srep37843 27897216 10.1038/srep37843 PMC5126664

[15] Wu HB, Guo CT, Lu RF, Xu LH, Wo EK, You JB, et al Genetic characterization of subtype H1 avian influenza viruses isolated from live poultry markets in Zhejiang Province, China, in 2011. Virus Genes. 2012;44(3):441–9. doi: 10.1007/s11262-012-0716-y 22252252 10.1007/s11262-012-0716-y

[16] Shortridge KF, Butterfield WK, Webster RG, Campbell CH. Isolation and characterization of influenza A viruses from avian species in Hong Kong. B World Health Organ. 1977;55(1):15–20. PMC2366618 302152

[17] Hoffmann E, Stech J, Guan Y, Webster RG, Perez DR. Universal primer set for the full-length amplification of all influenza A viruses. Arch Virol. 2001;146(12):2275–89. 11811679 10.1007/s007050170002

[18] Yu H, Hua RH, Zhang Q, Liu TQ, Liu HL, Li GX, et al Genetic evolution of swine influenza A (H3N2) viruses in China from 1970 to 2006. J Clin Microbiol. 2008;46(3):1067–75. doi: 10.1128/JCM.01257-07 18199784 10.1128/JCM.01257-07 PMC2268354

[19] Zhang P, Tang Y, Liu X, Liu W, Zhang X, Liu H, et al A novel genotype H9N2 influenza virus possessing human H5N1 internal genomes has been circulating in poultry in eastern China since 1998. J Virol. 2009;83(17):8428–38. doi: 10.1128/JVI.00659-09 19553328 10.1128/JVI.00659-09 PMC2738149

[20] Kang HM, Choi JG, Kim KI, Park HY, Park CK, Lee YJ. Genetic and antigenic characteristics of H4 subtype avian influenza viruses in Korea and their pathogenicity in quails, domestic ducks and mice. J Gen Virol. 2013;94(Pt 1):30–9. doi: 10.1099/vir.0.046581-0 23015746 10.1099/vir.0.046581-0

[21] Pu J, Wang S, Yin Y, Zhang G, Carter RA, Wang J, et al Evolution of the H9N2 influenza genotype that facilitated the genesis of the novel H7N9 virus. Proc Natl Acad Sci U S A. 2015;112(2):548 doi: 10.1073/pnas.1422456112 25548189 10.1073/pnas.1422456112 PMC4299237

[22] Li X, Shi J, Guo J, Deng G, Zhang Q, Wang J, et al Genetics, receptor binding property, and transmissibility in mammals of naturally isolated H9N2 Avian Influenza viruses. PLoS Pathog. 2014;10(11):e1004508 doi: 10.1371/journal.ppat.1004508 25411973 10.1371/journal.ppat.1004508 PMC4239090

[23] Yang H, Chen Y, Qiao C, He X, Zhou H, Sun Y, et al Prevalence, genetics, and transmissibility in ferrets of Eurasian avian-like H1N1 swine influenza viruses. Proc Natl Acad Sci U S A. 2016;113(2):392 doi: 10.1073/pnas.1522643113 26711995 10.1073/pnas.1522643113 PMC4720320

[24] Reed LJ, Muench H. A simple method of estimating fifty per cent endpoints. Am j Epidemiol. 1938;27(3).

[25] Hai-bo W, Chao-tan G, Ru-feng L, Li-hua X, En-kang W, Jin-biao Y, et al Characterization of a highly pathogenic H5N1 avian influenza virus isolated from ducks in Eastern China in 2011. Arch Virol. 2012;157(6):1131–6. doi: 10.1007/s00705-012-1259-1 22371029 10.1007/s00705-012-1259-1

[26] Treanor JJ, Snyder MH, London WT, Murphy BR. The B allele of the NS gene of avian influenza viruses, but not the A allele, attenuates a human influenza A virus for squirrel monkeys. Virology. 1989;171(1):1–9. 2525836 10.1016/0042-6822(89)90504-7

[27] He G, Qiao J, Dong C, He C, Zhao L, Tian Y. Amantadine-resistance among H5N1 avian influenza viruses isolated in Northern China. Antivir Res. 2008;77(1):72–6. doi: 10.1016/j.antiviral.2007.08.007 17897729 10.1016/j.antiviral.2007.08.007

[28] Hossain MJ, Hickman D, Perez DR. Evidence of expanded host range and mammalian-associated genetic changes in a duck H9N2 influenza virus following adaptation in quail and chickens. PloS One. 2008;3(9):e3170 doi: 10.1371/journal.pone.0003170 18779858 10.1371/journal.pone.0003170 PMC2525835

[29] Sun Y, Bi Y, Pu J, Hu Y, Wang J, Gao H, et al Guinea pig model for evaluating the potential public health risk of swine and avian influenza viruses. PloS One. 2010;5(11):e15537 doi: 10.1371/journal.pone.0015537 21124850 10.1371/journal.pone.0015537 PMC2990763

[30] Lowen AC, Mubareka S, Tumpey TM, Garcia-Sastre A, Palese P. The guinea pig as a transmission model for human influenza viruses. Proc Natl Acad Sci U S A. 2006;103(26):9988–92. doi: 10.1073/pnas.0604157103 16785447 10.1073/pnas.0604157103 PMC1502566

