# Supplementary material for: Isolation and characterization of H4N6 avian influenza viruses from mallard ducks in Beijing, China
Source: PLoS One. 2017 Sep 6;12(9):e0184437. doi: 10.1371/journal.pone.0184437 (PMC5587311; doi:10.1371/journal.pone.0184437)

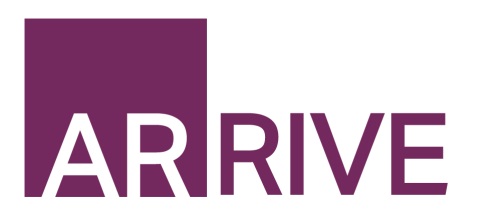


The ARRIVE Guidelines Checklist

Animal Research: Reporting In Vivo Experiments

Carol Kilkenny^1^, William J Browne^2^, Innes C Cuthill^3^, Michael Emerson^4^ and Douglas G Altman^5^

*^1^The National Centre for the Replacement, Refinement and Reduction of Animals in Research, London, UK, ^2^School of Veterinary Science, University of Bristol, Bristol, UK, ^3^School of Biological Sciences, University of Bristol, Bristol, UK, ^4^National Heart and Lung Institute, Imperial College London, UK, ^5^Centre for Statistics in Medicine, University of Oxford, Oxford, UK.*

|  | | ITEM | RECOMMENDATION | Section/ Paragraph |
| --- | --- | --- | --- | --- |
| 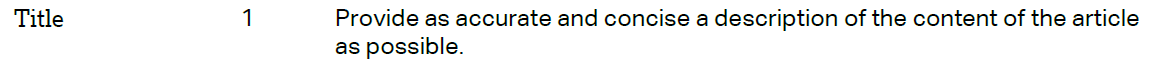 | | | Title |  |
| 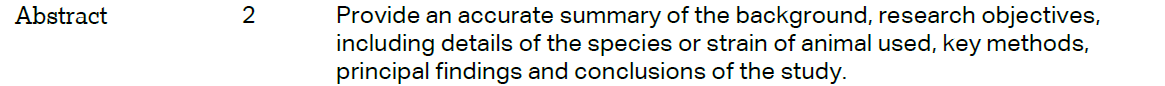 | | | Abstract |  |
| INTRODUCTION | | |  |  |
| 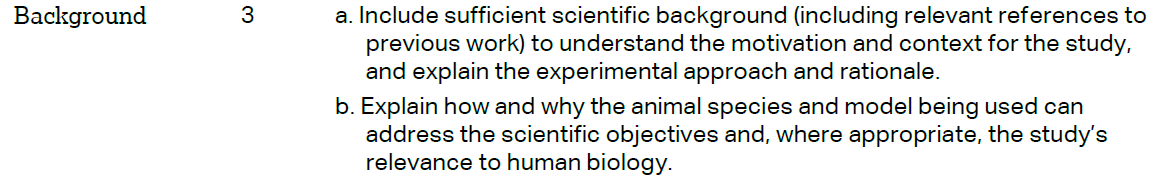 | | | Paragraphs 1-3  Paragraphs 2&3 |  |
| 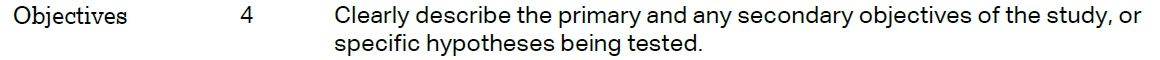 | | | Paragraph 3 |  |
| METHODS | | |  |  |
| 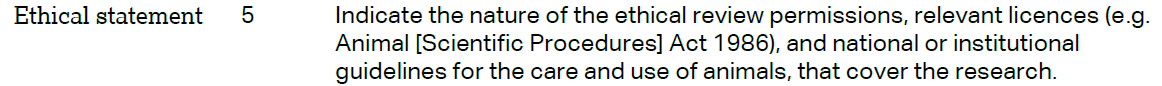 | | | Paragraph 1&2 |  |
| 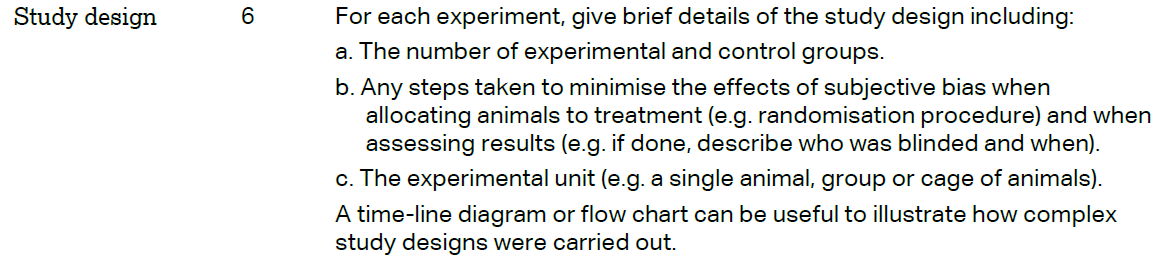 | | | Paragraphs 7-9 and  Figures 6-7 |  |
| 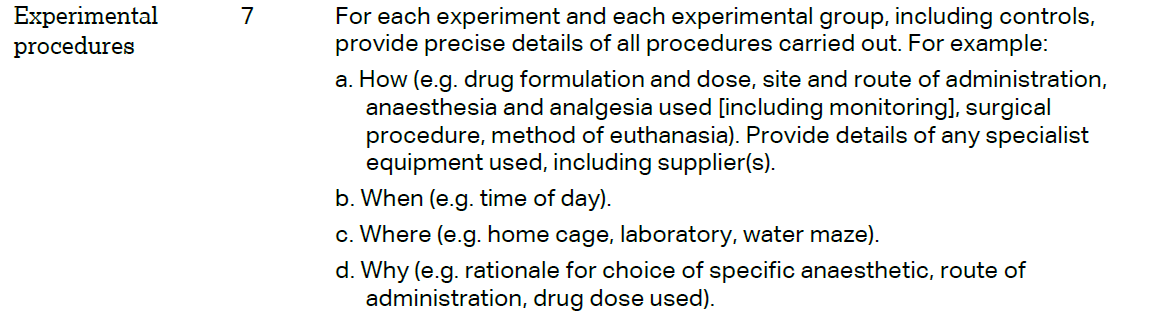 | | | Paragraphs 7-9 |  |
| 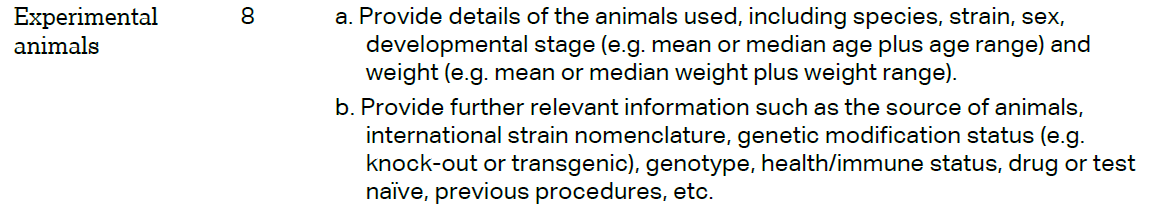 | | | Paragraphs 7&8 |  |

The ARRIVE guidelines. Originally published in *PLoS Biology*, June 2010^1^

| 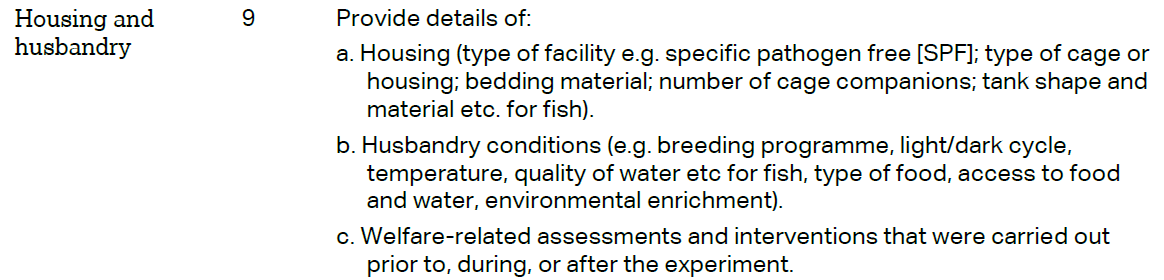 | Paragraphs 7-9 | |
| --- | --- | --- |
| 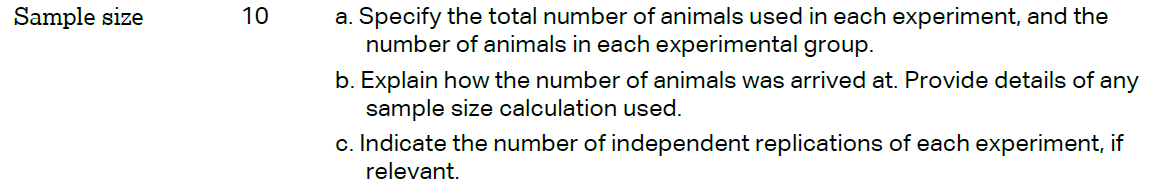 | Paragraphs 7-9 | |
| 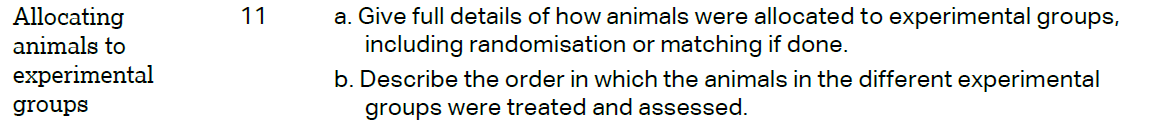 | Paragraphs 7-9 | |
| 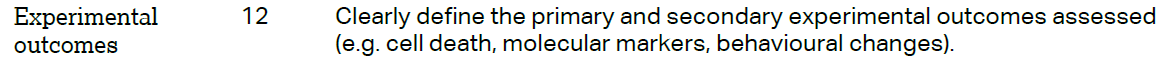 | Paragraphs 7-9 | |
| 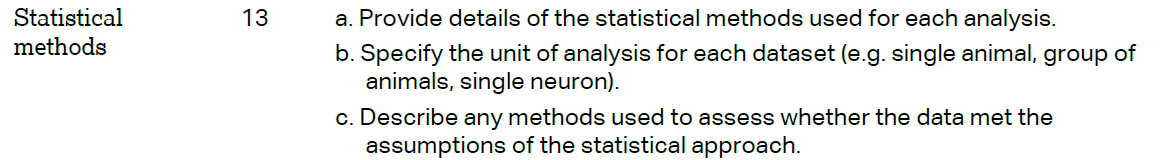 | Paragraph5 | |
| 5RESULTS |  | |
| 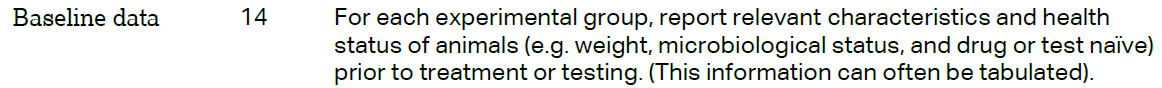 | Methods Paragraphs 7&8 | |
| 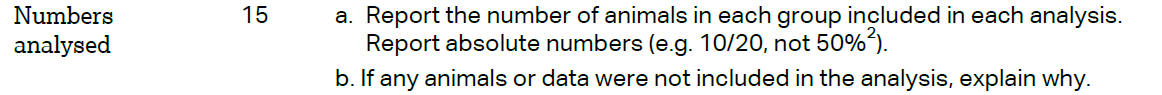 | Methods Paragraphs 7-9 and Figures 6&7 | |
| 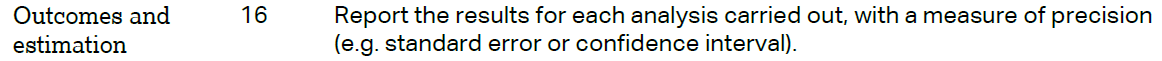 | Paragraphs 8-10 and Figures 6&7 | |
| 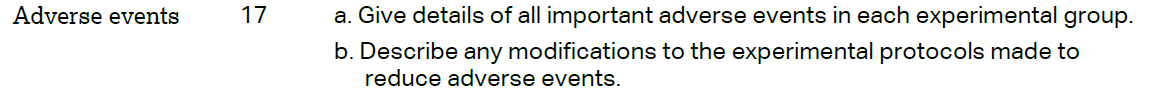 | N/A | |
| DISCUSSION |  | |
| 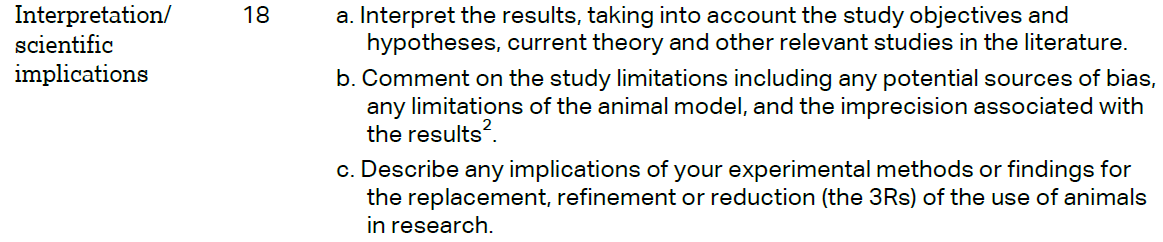 | Paragraphs 1-4 | |
| 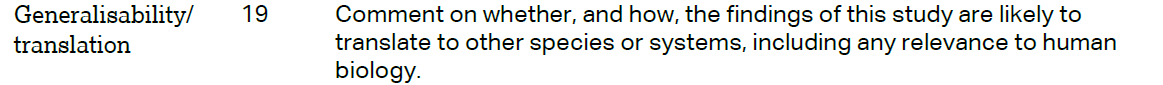 | Paragraph5&6 | |
| 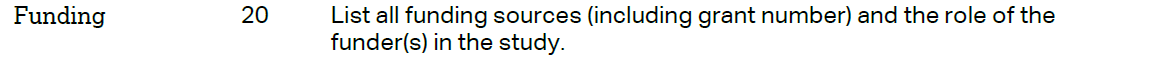 | | Submission form |


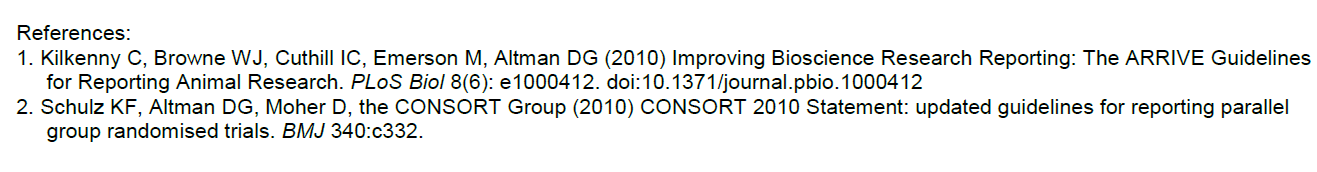

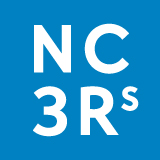

Supplement: S1 Checklist — (DOCX) [file pone.0184437.s001.docx]
