# Supplementary material for: Isolation and characterization of H4N6 avian influenza viruses from mallard ducks in Beijing, China
Source: PLoS One. 2017 Sep 6;12(9):e0184437. doi: 10.1371/journal.pone.0184437 (PMC5587311; doi:10.1371/journal.pone.0184437)
Supplement: S1 Table — (DOCX) [file pone.0184437.s002.docx]

| Strain | Abbreviation | PB2 | PB1 | PA | HA | NP | NA | M | NS | Genotype |
| --- | --- | --- | --- | --- | --- | --- | --- | --- | --- | --- |
| A/duck/Nanchang/4-165/2000 | 4-165 | 2 | 2 | 5 | 2 | 1 | 3 | 1 | 1 | 1 |
| A/mallard/ZhaLong/88/2004 | 88 | 2 | 2 | 5 | 2 | 1 | 2 | 1 | 1 | 2 |
| A/mallard/Yan chen/2005 | * | 1 | 2 | 1 | 2 | 1 | 3 | 1 | 1 | 3 |
| A/duck/Shanghai/Y20/2006 | Y20 | 1 | 3 | 6 | 2 | 1 | 3 | 1 | 1 | 4 |
| A/duck/Hunan/S1012/2009 | S1012 | 2 | 1 | 1 | 1 | 1 | 2 | 1 | 1 | 5 |
| A/duck/Anhui/S4155/2009 | S4155 | 1 | 1 | 1 | 1 | 4 | 2 | 1 | 1 | 6 |
| A/duck/Henan/S4179/2009 | S4179 | 2 | 1 | 1 | 2 | 1 | 1 | 4 | 1 | 7 |
| A/duck/Hunan/S1166/2010 | S1166 | 1 | 2 | 1 | 1 | 1 | 2 | 6 | 1 | 8 |
| A/duck/Henan/S1091/2010 | S1091 | 2 | 1 | 1 | 1 | 1 | 2 | 1 | 2 | 9 |
| A/duck/Guangxi/S1211/2010 | S1211 | 2 | 1 | 1 | 1 | 1 | 2 | 1 | 2 | 9 |
| A/duck/Guangzhou/S1167/2010 | S1167 | 2 | 1 | 1 | 1 | 1 | 1 | 1 | 1 | 10 |
| A/duck/Guangxi/S1107/2010 | S1107 | 2 | 1 | 1 | 1 | 4 | 2 | 1 | 2 | 11 |
| A/chicken/Hunan/S1248/2010 | S1248 | 1 | 1 | 1 | 1 | 1 | 1 | 1 | 1 | 12 |
| A/chicken/Hunan/S1267/2010 | S1267 | 1 | 1 | 1 | 1 | 1 | 1 | 1 | 1 | 12 |
| A/duck/Jiangsu/S2447/2011 | S2447 | 1 | 1 | 1 | 1 | 1 | 2 | 1 | 1 | 13 |
| A/duck/Fujian/S2169/2012 | S2169 | 2 | 1 | 1 | 1 | 1 | 2 | 1 | 1 | 5 |
| A/duck/Jiangxi/S2443/2012 | S2443 | 1 | 1 | 1 | 1 | 1 | 1 | 4 | 1 | 14 |
| A/duck/China/J1/2012 | J1 | 1 | 4 | 7 | 1 | 1 | 2 | 3 | 1 | 15 |
| A/duck/Hunan/S11090/2012 | S11090 | 2 | 1 | 2 | 1 | 1 | 2 | 1 | 1 | 16 |
| A/duck/Hunan/S11893/2012 | S11893 | 1 | 1 | 1 | 1 | 5 | 1 | 2 | 1 | 17 |
| A/duck/Anhui/S2193/2012 | S2193 | 2 | 2 | 1 | 1 | 1 | 2 | 5 | 1 | 18 |
| A/duck/Zhejiang/S2235/2012 | S2235 | 2 | 3 | 5 | 1 | 2 | 1 | 2 | 1 | 19 |
| A/duck/HuN/S11200/2012 | S11200 | 2 | 3 | 5 | 1 | 2 | 2 | 2 | 1 | 20 |
| A/duck/Zhejiang/S2088/2012 | S2088 | 2 | 2 | 5 | 1 | 2 | 2 | 2 | 1 | 21 |
| A/duck/Gugangxi/S2090/2012 | S2090 | 2 | 1 | 4 | 1 | 4 | 2 | 1 | 1 | 22 |
| A/duck/Zhejiang/D9/2013 | D9 | 2 | 3 | 3 | 1 | 3 | 2 | 2 | 1 | 23 |
| A/duck/Zhejiang/414/13 | 414 | 2 | 3 | 5 | 1 | 2 | 2 | 2 | 1 | 24 |
| A/duck/Zhejiang/413/13 | 413 | 2 | 2 | 5 | 1 | 2 | 2 | 2 | 1 | 25 |
| A/duck/Zhejiang/409/13 | 409 | 2 | 2 | 5 | 1 | 2 | 2 | 2 | 1 | 25 |
| A/duck/Zhejiang/D15/2013 | D15 | 2 | 2 | 5 | 1 | 2 | 2 | 2 | 1 | 25 |
| A/duck/Zhejiang/422/13 | 422 | 2 | 3 | 4 | 1 | 2 | 2 | 2 | 1 | 26 |
| A/duck/Zhejiang/418/13 | 418 | 2 | 2 | 5 | 1 | 3 | 2 | 2 | 1 | 27 |
| A/duck/Zhejiang/D2-1/13 | D2-1 | 2 | 2 | 5 | 1 | 3 | 2 | 2 | 1 | 27 |
| A/duck/Zhejiang/D14/2013 | D14 | 2 | 1 | 4 | 1 | 2 | 2 | 2 | 1 | 28 |
| A/mallard/Beijing/06/2016 | 6 | 1 | 1 | 1 | 1 | 1 | 1 | 1 | 1 | 29 |
| A/mallard/Beijing/10/2016 | 10 | 1 | 1 | 1 | 1 | 1 | 1 | 1 | 1 | 29 |
| A/mallard/Beijing/16/2016 | 16 | 1 | 1 | 1 | 1 | 1 | 1 | 1 | 1 | 29 |
| A/mallard/Beijing/25/2016 | 25 | 1 | 1 | 1 | 1 | 1 | 1 | 1 | 1 | 29 |

Table S1 H4N6 AIVs with different genotypes in China from 2000 to 2016.
